# The mitigating effect of low firearm background check requirements on firearm homicides in border states

**DOI:** 10.5249/jivr.vo113i2.1555

**Published:** 2021-07

**Authors:** Todd R. Ashworth, Claudia A. Kozinetz

**Affiliations:** ^*a*^ Department of Biostatistics and Epidemiology, College of Public Health, East Tennessee State University, USA.

**Keywords:** Firearm violence, Firearm homicide, Gun laws, Crime guns, Firearm injury

## Abstract

**Background:**

Firearm-related violence is a significant public health issue in the US. Research has found an increase in guns used in crimes sourced from low gun law states into high gun law states. The purpose of this study is to evaluate the effect of distance from states without universal background checks (UBC), background checks at shows (BCS), or permit to purchase (PTP) laws on firearm homicide rates in states with them.

**Methods:**

States were identified based on their enactment of laws that are designed to prevent the private sale of firearms to criminals. Demographic data for each county were obtained for the years 2014 through 2017. The border distance from a county in a state with the evaluated gun laws to the nearest border state without the gun laws was obtained using Google Maps. Multiple regression analyses were performed to test the relationship between border distance and firearm homicide rates.

**Results:**

The regression model evaluating all formats found the border distance was negatively associated with firearm homicides (p=.009). The parameter estimate indicated as border distance increased, the firearm homicide rate decreased. When counties with UBC or PTP on all guns were evaluated separately from all formats model, the statistical significance was lost (p=.62). In counties where all handgun sales either require a background check or a PTP is required, the distance was also not statistically significant (p=.11).

**Conclusions:**

This study provides evidence that there may be a mitigating effect on the reduction of firearm homicides in states that require background checks or PTP on private sales when there is a state in close proximity that did not have these laws. Limited counties at certain distances may have contributed to the insignificant findings in other models.

## Introduction

Firearm-related violence is a significant public health issue in the United States. According to The Centers for Disease Control and Prevention (2018), the number of firearm deaths surpassed motor vehicle accident-related deaths for the first time in recent history with 39,773 compared to 38,659 deaths, respectively.^1^ During the years 2014 through 2017, firearm mortality was the 14th leading cause of death for all age groups and the second leading cause of death for ages 15 through 34, contributing to substantial years of potential life lost.^1^ In addition to the cost of human life, firearm-related injuries have a substantial economic cost. Inpatient services for firearm-related injuries are estimated to cost approximately $2.8 billion annually, and total societal costs are estimated to be $174 billion annually.^2,3^

A significant portion of firearm-related deaths is due to interpersonal violence. During the years 2014 through 2017, approximately 73% of all homicides involved firearms, and nearly 36% of all firearm deaths were homicides.^1^ Over the years, multiple laws have been enacted to mitigate mortality and injury from firearm use. Consistently, states with stricter gun legislation show lower firearm homicide rates.^4^ Jehan et al. (2018) estimated a 28% decrease in firearm-related injuries in states with strict firearm legislation.^5^ Among the laws studied, those that expand background checks appear to decrease firearm homicide most consistently.^4^ Laws that expand background checks include Universal background checks (UBC), permit to purchase or license (PTP), which includes a background check, and background checks at gun shows (BCS). UBCs have been estimated to account for a 14.9% reduction in overall homicide rates.^6^ When Missouri repealed their PTP law for handguns, firearm homicide increased 34% in the first year and 24.9% in the three years following the repeal.^7^ PTP laws were found to decrease crime-guns original purchased in the same state by 3.9%.^8^ Effective gun legislation has the potential to prevent injury and mortality from firearms use. 

In states where there is strict gun legislation, research has found an increase in guns used in crimes sourced from low gun law states into high gun law states.^8,9^ Out-of-state crime guns in high gun law states are generally imported from low gun law states.^10^ Kahane (2013) found PTP for handgun purchases increased gun imports by 33.5%.^10^ Webster (2001) found a 37.1% decrease in in-state purchased crime guns in states with a PTP law and registration law. The ease of access outside the retail market appears to be the mechanism by which PTP, UBC, and BCS work.^11^ In states without these laws, individuals can purchase guns via private sales without checks or other safeguards that would generally prevent criminals from purchasing guns. According to the United States Bureau of Alcohol, Tobacco, Firearms, and Explosives (ATF) (2002), 85% of guns used in crimes are recovered from someone other than the original purchaser.^12^ In states where these types of transactions are regulated, it is more difficult to obtain a firearm. In a 2017 study, 41% of surveyed criminals reported having more difficulty getting a handgun after Maryland enacted a PTP in 2013, citing increased cost, lack of trusted sources, or people less willing to engage in straw purchases on their behalf.^13^

One facet affecting the underground firearm market may be the distance that needs to be traveled to reach a state where a gun can be obtained more easily. Coates and Pearson-Merkowitzz (2017) found that the size of a state was associated with lower out-of-state crime guns recovered.^9^ Further, Webster (2001) found a small relationship between out-of-state crime guns and proximity to states with minimal restrictions on gun purchases.^11^ In most cases, states with UBC, PTP, or BCS border another state without these laws. According to the ATF (2018), most out-of-state guns recovered in crimes came from nearby or border states.^14^ To date, there has been little research evaluating the distance a location is from a state with minimal firearm purchase regulations and the impact on firearm homicides. Kaufman, Morrison, Branas, and Wiebe (2018) attempted to evaluate the impact of distance on firearm homicide and suicide using a degrading policy score and did not find a significant relationship.^15^ This study, however, did not specifically isolate states with UBC, PTP, or BCS; scoring was built around UBC, PTP, and four other laws. Matthay et al. (2017) evaluated the relationship between short-term increased firearm deaths and injuries in California following gun shows in California and Nevada and geographic ranges of 60-minute, 120-minute, and 180-minute drive times.^16^ The study found that there was an increase in firearm deaths in the California geographic locations following gun shows in Nevada that did not have a UBC during the study timeframe. Evaluation of drive times was null but low numbers of observations at the two higher distances might have driven the null results. The purpose of this study is to evaluate the effect of distance from states without any additional UBC, BCS, PTP laws in private sales on firearm homicide rates in states with them. Additionally, this study will attempt to determine if there is an effect in states where the additional laws apply to handgun sales, all gun sales, or where the additional checks are for gun show sales. Results could help inform a national or state-level decision on whether to add additional background check requirements for gun purchases.

## Methods 

**Regulations**

States were first identified based on their enactment of specific gun laws by the year 2014. The year 2014 was selected to provide significant time for the laws to have their desired effect. The gun laws and associated states were identified from the database at www.statefirearmlaws.org, initially built by Siegal et al. (2021), which is an ongoing project to categorize 134 different gun laws across all 50 states in the United States.^17,18^ Gun law data is available from 1991 through 2020. The following laws were used for selection with the statefirearmlaws.org database code in parentheses.^18^

• Universal background checks at the point of purchase of all guns including private sales, gun shows and retail purchases (universal)

• Universal background checks at the point of purchase of all handguns including private sales, gun shows and retail purchases (universal)

• Background checks required at the point of purchase at gun shows for all guns (gun show)

• Background checks required at point of purchase at gun shows for all handguns (gun show)

• Required permit which includes a background check for the purchase of all guns (universal permit)

• Required permit which includes a background check for the purchase of all handguns (universal permit)

Counties within states that had had universal background checks or universal permits for all guns were coded as a 1. Counties within states that had universal background checks or universal permits for handguns were coded as a 2. Counties within states with background checks for gun shows but not private sales for all guns were coded as a 3. All counties within states that did not have any of these laws were coded as a 0. 

From the 48 contiguous states, 332 counties were selected from the CDC’s WONDER database (2018).^1^ The selected counties had to have an average of 100,000 residents for the years 2014 through 2017, and the firearm homicide rate could not be listed as “unreliable.” Firearm homicides and total firearm homicides for the years 2014 through 2017 were obtained from the CDC’s WONDER database for each county. 

**Demographics**

Demographic variables for this study were based on the associated literature. Literature has found consistent associations between homicide rates and poverty. Additionally, consistent associations between firearm homicides and the proportion of Black/African Americans in communities have been reported. Siegel, et al. (2019) found a significant predictive relationship between firearm homicides and violent crime after subtracting firearm homicides.^6^ To control for this recently found predictor of firearm homicides, the adjusted violent crime data is included in the linear regression model. The adjusted violent crime rate was calculated by subtracting firearm homicides from the violent crime total and then calculating the rate per 100,000 population.

Demographic data for each county was obtained from the US Census Bureau’s “Fact Finder” for the years 2014 through 2017.^19^ Demographic confounders include race and poverty. Violent crime data sets were obtained from www.countyhealthrankings.org, which contains consolidated violent crime data for each county from the FBI Uniform Crime Report.^20^

**Border Distance**

The border distance from a county in a state with the listed gun laws to the nearest border state without the listed gun laws enacted by 2014 was obtained using Google Maps.^21^ The border distance was measured from the center of a county as estimated by Google Maps to the nearest state line that did not have any of the gun laws. For example, Illinois borders Wisconsin, Indiana, Missouri, and Kentucky, which do not have the additional private purchase requirement laws. Cook County in Illinois was measured from the center of the county to the nearest state line that does not have these laws, which was Indiana. The distance was measured using highway or freeway travel. The distance was divided into 10-mile segments. If a distance did not fall on a 10-mile mark, it was moved up to the nearest mark. For example, if the distance was 181 miles, it was moved up to the 190-mile mark. In all cases, there was a town or city at or near the border. 

**Data Analysis**

Multiple linear regression models were generated using the data for each county. Only counties with BCS, UBC, PTP were used in the regression to prevent spurious findings due to higher firearm homicide rates in states without these laws. The outcome variable was firearm homicide rates. The predictor variables were the border distance in 10-mile segments, adjusted violent crime rate (violent crime minus firearm homicides), the percent of the Black/African American populations, and the proportion of the populations that are in poverty. Additional regression analyses were done individually for states where the laws only apply to handgun sales, for states where the laws apply to all gun sales, and for states where the only additional law is for gun shows. The data analysis for this paper was generated using SAS studio software, version 3.8. Copyright © 2021 SAS Institute Inc. SAS and all other SAS Institute Inc. product or service names are registered trademarks or trademarks of SAS Institute Inc., Cary, NC, USA.^22^

The natural log of the homicide rate was used to counter violations of the homoscedasticity assumption. All regressions used studentized residuals greater than 2.0 to eliminate influencing outliers. Variance inflation factor, conditional index, and tolerance tests were conducted to check for complications due to collinearity. 

## Results

Of the 332 counties, 184 were in states without additional background checks regulating private sales. There were 87 counties in states that required background checks or a permit to purchase on all gun purchases. There were 55 counties in states that required background checks or a permit to purchase on all handgun purchases. Six counties were in states that required background checks for all firearm purchases made at gun shows. Descriptive statistics for counties are provided in Table 1.

**Table 1:** Descriptive statistics of Counties used in this study.

	no additional background checks laws (n=184)	All formats (n=148)	All guns (n=87)	Handguns (n=55)	Gun shows (n=6)
	Mean (STD) (Range)	Mean (STD) (Range)	Mean (STD) (Range)	Mean (STD) (Range)	Mean (STD) (Range)
Population (avg. popultion 2014-2017)^a^	508023.14	765770.65	975877.4	469990.24	430543.25
	(599778.40)	(1041246.25)	(1284915.54)	(379856.75)	(233014.22)
	(103721.25 to 4555576.50)	(108129.75 to 10147104.75)	(110466.75 to 10147104.75)	(124227.50 to 1756780.25)	(108129.75 to 793581.75)
Firearm Homicides (per 100,000 population) ^b^	6.38	4.19	3.58	5.36	2.37
	(5.1)	(4.03)	(2.55)	(5.58)	(1.77)
	(.7 to 40.7)	(.4 to 35.2)	(.4 to 13.1)	(.8 to 35.2)	(0.9 to 5.8)
Adjusted Violent Crime Rate (per 100,000 population) ^c^	436.93	399.74	408.37	407.29	205.49
	(235.35)	(213.72)	(182.85)	(255.22)	(131.15)
	(62.58 to 1873.03)	(88.12 to 1425.61)	(88.12 to 845.49)	(148.13 to 1425.61)	(89.78)(445.6)
% in Poverty	15.69	14.06	13.86	14.35	14.32
	(4.76)	(4.82)	(4.76)	(5.08)	(3.89)
	(5.7 to 31.3)	(6.3 to 30.1)	(6.3 to 29.6)	(6.5 to 30.1)	(9 to 18.1)
% Black or African American	19.67	12.37	9.55	17.99	1.76
	(14.84)	(11.24)	(7.77)	(13.78)	(1.81)
	(.27 to 71.56)	(.42 to 62.33)	(.68 to 38.78)	(2.31 to 62.33)	(.42 to 5.32)
Border Distance (in 10-mile segments)	0	13.07	14.93	7.4	38.17
		(9.28)	(8.17)	(4.4)	(3.31)
		(2 to 44)	(2 to 36)	(2 to 18)	(34 to 44)

a Calculated from the total population from January 2014 to December 2017. b Calculated from the total count of firearm homicides from January 2014 to December 2017. c Calculated from the total count of violent crimes minus firearm homicides from January 2014 to December 2017.

In the initial regression model (Table 2) evaluating all formats, the border distance was negatively associated with firearm homicides (p=.009). The parameter estimate was approximately negative .01, indicating as border distance increases, the firearm homicide rate decreases. When evaluating counties in states where all gun purchases require background checks or PTP, the border distance was not statistically significant (p=.62). Border distance was not statistically significant in states where all handgun purchases required background checks (p=.11). Counties that only required additional background checks or PTP at gun shows could not be evaluated due to only six counties meeting the criteria. No collinearity complications were detected in the Variance inflation factor, conditional index, or tolerance tests. A correlation matrix is provided in Table 3.

**Table 2:** Parameter values evaluating background check formats predicting firearm homicides.

	All Formats		All Guns		Handguns	
	Parameter Estimates (95% Confidence Interval)	Standard Error	Parameter Estimates (95% Confidence Interval)	Standard Error	Parameter Estimates (95% Confidence Interval)	Standard Error
**Intercept**	-.105 (-.34 to .13)	.12	-.437* (-.806 to -.068)	.185	.164 (-.177 to .505)	.169
**Border Distance**	-.01** (-.018 to -.003)	.004	-.003 (-.015 to .009)	.006	-.018 (-.039 to .004)	.01
**Adjusted Violent Crime Rate**	.001** (.001 to .002)	<.001	.001** (.0005 to .002)	<.001	.001** (.0006 to .002)	<.001
**% Black of African American**	.018** (.01 to .025)	.004	0.026** (.012 to .04)	.026	.013* (.003 to .022)	.005
**% in Poverty**	.053** (.036 to .07)	.009	.06** (.036 to .087)	.061	.043** (.02 to .066)	.011
**Adj. R Square**	.71		.625		.793	
**Homoscedasticity (White test)**	p=.196		p=.683		p=.381	
**No. of Observations**	140		81		53	

*p<.05 **p<.01 Parameters are calculated from the Natural log of firearm homicides

**Table 3:** Correlation matrix for predictor and outcome variables (n=148).

	Firearm Homicide Rate	Border Distance (10-mile Segments	%Black or African American	% in Poverty	Adjusted Violent Crime Rate
**Firearm Homicide Rate**	1	-.192*	.637**	.526**	.733**
**Border Distance (10-mile Segments)**	-.192*	1	-.385**	-.006	-.189*
**%Black or African American**	.637**	-.385**	1	.263**	.584**
**% in Poverty**	.526**	-.006	.263**	1	.579**
**Adjusted Violent Crime Rate**	.733**	-.189*	.584**	.579**	1

*p<.05 **p<.01 Correlation matrix is calculated using counties not in states without additional background check requirements.

## Discussion

This study provides evidence that there may be a mitigating effect on the reduction of firearm homicides in states that require background checks or PTP on private sales when there is a state in close proximity that does not have these laws. This relationship is particularly evident in the models that combine all formats. The multiple linear regression models for all formats together found reductions in firearm homicide rates as border distance increased independently of poverty, race, and adjust violent crime rates. Based on the regression model's findings evaluating all formats, Cook county, which contains Chicago, would have seen a reduction of approximately .6 firearm homicides per 100,000 residents if Wisconsin and Indiana, two states within 100 miles, had one of the additional laws during the years 2014 through 2017. Over the four-year span, this would amount to about 120 fewer firearm homicides. These findings are further supported by Coates and Pearson-Merkowitzz (2017) and Webster’s (2001) findings regarding crime guns and the size of a state, and the distance. ^9,11^ Counties along the border may have easier access to firearms. For example, within 80 miles of distance, a county could more easily drive out of state and make purchases in a garage sale without a background check and is not as reliant on the underground market. Counties that are at greater distances not only face a significant distance barrier, but the underground market is also likely to be more expensive due to reduced availability. 

Limited counties at certain distances may have contributed to the insignificant findings in other models. The majority of counties in the models that evaluated all guns were beyond 80 miles; only 17 were within 80 miles. The handgun model only had one county 180 miles or further, likely a result of the majority of these states being smaller and very close to states without an additional background check or PTP law. Adding substantial more counties may have resulted in more clear findings. 

Further research is recommended into the mitigating effect of states without additional background checks or PTP laws on states with them. Additional research could include effects on firearm-related injuries, overall homicide rates, overall violent crime, or costs associated with firearm-related deaths. Such research would be valuable information for consideration in adding laws that regulate private firearm sales.

## References

[1] Centers for Disease Control and Prevention, National Centers for Health Statistics. Underlying Cause of Death 1999-2019 on CDC WONDER Online Database, 2021, http://wonder.cdc.gov/ucd-icd10.html, accessed 25 March 2021.

[2] Gani F, Sakran JV, Canner JK (2017). Emergency department visits for firearm-related injuries in the United States, 2006-14. Health Aff (Millwood)..

[3] Miller T. The cost of firearm violence. Children’s Safety Network, 2012, https://www.childrenssafetynetwork.org/publications/cost-firearm-violence, accessed March 26, 2021.Alphabet Inc. Google Maps. https://www.google.com/maps, 2019, accessed 17 July 2019.

[4] Lee LK, Fleegler EW, Farrell C, Avakame E, Srinivasan S, Hemenway D (2017). Firearm laws and firearm homicides: A systematic review. JAMA Intern Med.

[5] Jehan F, Pandit V, O’Keeffe T, Azim A, Jain A, Tai SA (2018). The burden of firearm violence in the United States: stricter laws result in safer states. J Inj Violence Res.

[6] Siegel M, Pahn M, Xuan Z, Fleegler E, Hemenway D (2019). The Impact of State Firearm Laws on Homicide and Suicide Deaths in the USA, 1991–2016: a Panel Study. J Gen Intern Med.

[7] Webster D, Crifasi CK, Vernick JS (2014). Effects of the repeal of Missouri’s handgun purchaser licensing law on homicides. J Urban Health.

[8] Collins T, Greenberg R, Siegel M, Xuan Z, Rothman EF, Shea WC (2018). State Firearm Laws and Interstate Transfer of Guns in the USA, 2006 – 2016. J Urban Health.

[9] Coates M, Pearson-Merkowitzz S (2017). Policy Spillover and Gun Migration: The Interstate Dynamics of State Gun Control Policies. Soc Sci Q.

[10] Kahane LH (2013). Understanding the interstate export of crime guns: A gravity model approach. Contemp Econ Policy.

[11] Webster DW, Vernick J, Hepburn L (2001). Relationship between licensing, registration, and other gun sales laws and the source state of crime guns. Inj Prev.

[12] United States Bureau of Alcohol Tobacco Firearms and Explosives. Crime Gun Trace Reports: The Youth Gun Interdiction Initiative. Washington D.C.; 2002.

[13] Crifasi CK, Buggs SAL, Choksy S, Webster DW (2017). The initial impact of Maryland’s firearm safety act of 2013 on the supply of crime handguns in Baltimore. Russell Sage Found J Soc Sci.

[14] United States Bureau of Alcohol Tobacco Firearms and Explosives. Firearm Trace Data - 2017. 2019, https://www.atf.gov/resourcecenter/firearms-trace-data-2017, accessed 28 March 2021.

[15] Kaufman EJ, Morrison CN, Branas CC, Wiebe DJ (2018). State Firearm Laws and Interstate Firearm Deaths From Homicide and Suicide in the United States A Cross-sectional Analysis of Data by County. JAMA Intern Med.

[16] Matthay EC, Galin J, Rudolph KE, Farkas K, Wintemute GJ, Ahern J (2017). In-State and interstate associations between gun shows and firearm deaths and injuries. Ann Intern Med.

[17] Siegel M, Pahn M, Xuan Z, Ross CS, Galea S, Kalesan B (2017). Firearm-Related Laws in All 50 US States, 1991-2016. Am J Public Health.

[18] Siegel M. State Firearm Laws: National Data. http://www.statefirearmlaws.org/national-data, 2021, accessed 27 March 2021.

[19] United States Census Bureau. American Fact Finder, 2017, https://factfinder.census.gov, accessed 16 February 2019.

[20] University of Wisconsin Population Health Institute, Robert Wood Johnson Foundation. County Health Rankings 2021, https://www.countyhealthrankings.org/explore-health-rankings/rankings-data-documentation/national-data-documentation-2010-2017, accessed 28 March, 2021.

[21] Alphabet Inc. Google Maps. https://www.google.com/maps, accessed July 17, 2019.

[22] SAS Institute Inc. Statistical Analysis System Studio Version 3.8. 2021, https://www.sas.com/en_us/software/studio.html, accessed 9 March 2021.

